# Efficacy and safety of sublingual immunotherapy for allergic rhinitis: A network meta-analysis

**DOI:** 10.3389/fimmu.2023.1144816

**Published:** 2023-03-30

**Authors:** Zao Ji, Feifei Jiang

**Affiliations:** Department of Otorhinolaryngology, The First Hospital of China Medical University, Shenyang, Liaoning, China

**Keywords:** allergic rhinitis, sublingual immunotherapy, network meta-analysis, clinical efficacy, safety

## Abstract

**Background:**

To systematically evaluate the clinical efficacy and safety of sublingual immunotherapy for allergic rhinitis (AR) and provide evidence for clinical treatment.

**Methods:**

A literature search was performed on the China National Knowledge Infrastructure (CNKI), Wanfang database, PubMed, Web of Science, Cochrane Library, and Embase database. Data from randomized controlled trials (RCTs) of sublingual immunotherapy for AR were screened and extracted from the establishment of those databases to November 2022. Subsequently, a network meta-analysis was performed using a statistical software R 4.2.

**Results:**

Totally 22 RCTs that met the inclusion and exclusion criteria and screened from 1,164 literature were included. A total of 4,941 AR patients were involved in the 22 trials, as well as five interventions including placebo, pharmacotherapy, subcutaneous immunotherapy_dust mite, sublingual immunotherapy_dust mite, and sublingual immunotherapy_ grass mix plus pollen extract. The results of network meta-analysis showed that, based on symptom scores after different interventions for AR, the most effective treatments for AR were in order as follows: sublingual immunotherapy_dust mite, subcutaneous immunotherapy_dust mite, sublingual immunotherapy_ grass mix plus pollen extract, placebo, and pharmacotherapy. Importantly, sublingual immunotherapy had fewer adverse reactions and higher safety.

**Conclusion:**

Sublingual immunotherapy_dust mite for AR has the best efficacy, whereas traditional medicine has the worst. More high-quality studies with a large sample and multiple centers are needed to verify this conclusion in the future, so as to further provide more reliable evidence-based medical evidence for the clinical treatment options of AR patients.

## Introduction

Allergic rhinitis (AR) is a common disease in otorhinolaryngology that occurs in atopic individuals after exposure to allergens. It is a non-infectious, chronic inflammatory disease of the nasal mucosa mediated mainly by immunoglobulin E (IgE) (1). Worldwide, AR affects more than 500 million people, with a prevalence of 10%–40%. In China, the prevalence is about 4%~38%, and its incidence is increasing year by year (2, 3). AR is categorized into seasonal AR and perennial AR based on the types of allergens. Seasonal AR is mainly caused by seasonal inhalant allergens such as pollen and fungi, whereas perennial AR is mainly triggered by perennial inhalant allergens such as dust mites, cockroaches, and animal dander. The main clinical manifestations of AR are paroxysmal sneezing, nasal congestion, nasal itching, and runny nose. AR patients with pollen allergy are often accompanied by ocular symptoms such as eye itching, lacrimation, eye redness, and burning sensation; AR patients with bronchial asthma may be accompanied by pulmonary symptoms such as wheezing, cough, shortness of breath, and chest tightness; and some AR patients also experience psychological disorders like anxiety and depression (4, 5).

At present, the main methods commonly used to treat AR in clinical practice are environmental control, pharmacotherapy, immunotherapy and surgical treatment. As the only therapy considered to change the natural course of allergic diseases, specific immunotherapy (SIT) is targeted at the etiology of IgE-mediated type I allergic disorders. Specifically, SIT induces immune tolerance through allergen extracts and then improves the symptoms of AR patients when they are reexposed to allergens (6). SIT can be classified into sublingual immunotherapy (SLIT) and subcutaneous immunotherapy (SCIT) according to different routes of administration. SCIT, as the gold standard, occupies an important part in the AR treatment. However, SLIT has gradually become a safe and effective immune alternative with the promotion and wide application of SLIT in clinical treatment, but there are still some controversies about the choice of the two treatment methods (7). In addition, most current clinical studies for AR treatment focus on the efficacy comparison between placebo and active drugs, with few comparisons among immunotherapies. Due to the differences in SIT procedures, as well as safety and efficacy among different routes of administration, different immunotherapies still face various selection challenges in clinical application. Therefore, the aim of this study was to investigate the clinical efficacy and safety of SLIT in the treatment of AR. Moreover, a network meta-analysis was conducted on randomized controlled trials (RCTs) of SLIT and SCIT in the treatment of AR, which compared the symptom scores and adverse reactions of different interventions in the treatment of AR. It is hoped that this study will provide more evidence-based clinical evidence and data in support of optimizing the clinical treatment of AR, so as to select a better treatment strategy to guide clinical medication.

## Materials and methods

### General information

The published clinical studies on SIT in the treatment of AR before November 2022 were collected, collated, and analyzed to systematically evaluate the efficacy and safety of SLIT in the treatment of AR. This study followed the Preferred Reporting Items for Systematic Evaluation and Meta-Analysis (PRISMA) guidelines for analysis (8).

## Method

### Database and literature search strategy

The adopted databases included the China National Knowledge Infrastructure (CNKI), Wanfang database (Wanfang), PubMed, Web of Science, Cochrane Library, and Embase database. Subject terms such as “immunotherapy” and “allergic rhinitis” were combined and searched from database establishment to November 2022, and then relevant clinical studies were screened one by one.

### Inclusion criteria

The inclusion criteria are as follows (1): study design—RCT studies are published at home and abroad; (2) study objects—patients clinically diagnosed with AR; (3) intervention measures—placebo, pharmacotherapy alone (PharT, including corticosteroids and antihistamines), subcutaneous immunotherapy_dust mite (SCIT_DM), sublingual immunotherapy_dust mite (SLIT_DM), and sublingual immunotherapy_grass mix plus pollen extract (SLIT_GP); (4) outcome measures—symptom scores (standard 4 scale: 0, no symptoms; 1, mild symptoms, not causing discomfort or interfering with daily life; 2, moderate symptoms, causing discomfort but not interfering with daily activities/sleep; 3, severe symptoms, causing intense discomfort and interfering with daily life/sleep and adverse reactions (local or systemic adverse reactions).

### Exclusion criteria

The exclusion criteria are as follows: (1) The study design of literature is not satisfying. (2) The intervention measures: SIT is not involved in the experimental group of the research literature. (3) The diagnostic criteria or outcome measures are not clear. (4) The key data required for this network meta-analysis are not provided in the literature, and no relevant data are obtained after contacting the original author. (5) The quality of the literature is poor, or important data are missing. (6) The literature is repeatedly reported, or the original text cannot be obtained. (7) The literature is a single-arm study, a retrospective study, a case report, a systematic review, a meeting transcript, review, or an animal experimental study.

### Literature screening and data extraction

The bibliography information retrieved from each database was imported into EndNote X8 software, and information supplement and literature duplicate checking were performed by combining manual operation with software. Subsequently, two investigators read the original text to screen the literature based on the inclusion and exclusion criteria. After cross-checking their statistical results, they extracted the relevant clinical data and outcome measures from the literature. If one of them disputes the screened literature results, a third investigator will arbitrate the dispute and decide the final result. Finally, the investigators gathered the original literature and extracted the literature title, author, publication time, study design type, basic characteristics of the study object, intervention measures, outcome indicators, and other relevant data.

### Statistical analysis

The network meta-analysis of various interventions was performed using packages such as “gemtc”, “metafor”, and “meta” of R 4.2 software. This analysis module is a multivariate meta-analysis model based on Bayesian models. Odds ratio (OR) and 95% confidence intervals (CI) were used to represent binary variables, and standardized mean difference (SMD) and 95% CI to represent continuous variables. Statistical heterogeneity among different studies was evaluated using the *χ^2^* test. The fixed effect model was selected for meta-analysis if *P* > 0.1 and *I* ^2^ < 50%, which indicated that there was no significant difference in the heterogeneity test; that is, homogeneity existed among the included studies. If *P* < 0.1, *I* ^2^ ≥ 50%, significant heterogeneity existed among the included studies, and a random-effect model was chosen for meta-analysis and subgroup analysis and sensitivity analysis were performed on possible causes of heterogeneity. Additionally, the probability of the overall response rate of various interventions was ranked in terms of the surface under the cumulative ranking area (SUCRA). The results of SUCRA were presented as percentages. In particular, comparisons were carried out between each intervention and an imaginary intervention with a very high response rate to simulate the percentage of those interventions that would achieve the optimal intervention. The larger the percentage of SUCRA of an intervention, the better the treatment outcome.

## Results

### Literature search results and basic characteristics of the included studies

**Figure 1:**
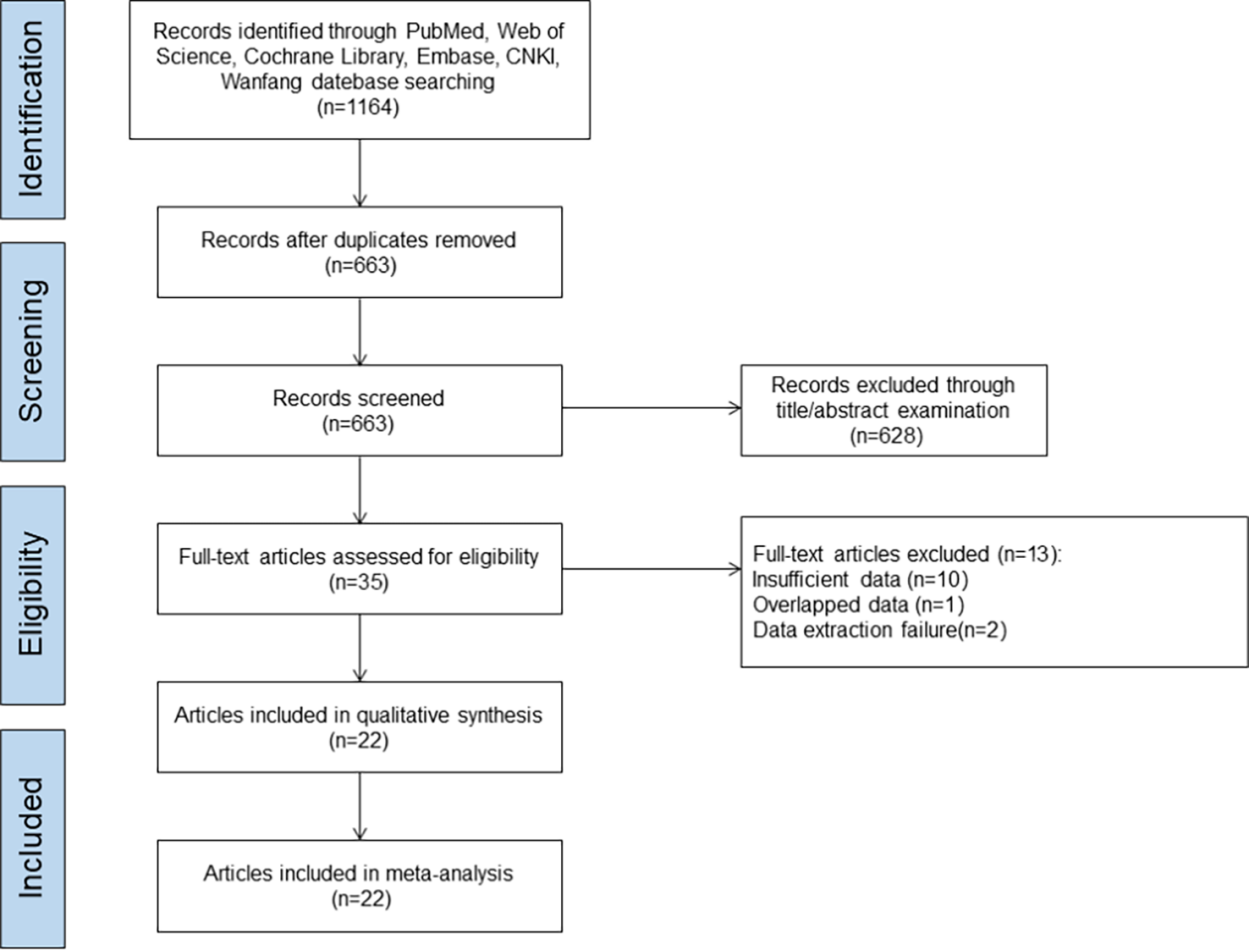
Literature screening flowchart.

A total of 1,164 literature was initially screened from the databases involved in this study. The literature screening procedure is shown in **Figure 1**. After excluding duplicates, 663 articles remained; after the investigators browsed titles and abstracts of the literature, 628 articles were excluded; following reading the full-text articles, 13 articles that did not meet the inclusion criteria were excluded; finally, 22 literature were included (9–30). A total of 4,941 AR patients were included in the literature study in this analysis, with 2,440 patients in the test group (SLIT_DM group and sublingual SCITGP group) and 2,501 patients in the control group (placebo group/PharT group/SCIT_DM group). The detailed basic characteristics of the included literature are shown in **Table 1**.

**Table 1:** Basic characteristics of the included literature.

Included literature	Sample size (n)	Interventions	Follow-up time (month)	Outcome indicator
Moreno-Ancillo (2007)	53/52	Placebo/SLIT-GP	84.2/79.1	①②
Bozek (2012)	57/51	Placebo/SLIT-DM	36/36	①②
Eifan (2009)	16/16/16	PharT/SLIT-DM/SCIT-DM	28.71/24.75/30.4	①②
Didier (2007)	156/160	Placebo/SLIT-GP	4/4	①②
Bufe (2008)	127/126	Placebo/SLIT-GP	12/12	①②
Stelmach (2012)	18/18	Placebo/SLIT-GP	24/24	②
Bergmann (2013)	163/150	Placebo/SLIT-DM	12/12	①②
Worm (2014)	261/275	Placebo/SLIT-GP	24/24	②
Demoly (2016)	338/336	Placebo/SLIT-DM	4/4	②
Guez (2000)	36/36	Placebo/SLIT-DM	24/24	①②
Durham (2005)	136/141	Placebo/SLIT-GP	4.5/4.5	①②
Yonekura (2010)	9/19	Placebo/SLIT-DM	10/10	②
Yonekura (2021)	257/255	Placebo/SLIT-GP	10/10	①②
Biedermann (2019)	314/320	Placebo/SLIT-GP	9.7/9.7	②
Okamoto (2018)	217/205	Placebo/SLIT-DM	12/12	②
Ma (2014)	60/60	Placebo/SLIT-DM	12/12	①②
Nong (2014)	28/28	Placebo/SLIT-DM	24/24	①
Qian (2017)	45/45	SLIT-DM/SCIT-DM	12/12	①②
Wang (2015)	48/79	SLIT-DM/SCIT-DM	12/12	②
Zhao (2020)	31/30	SLIT-DM/SCIT-DM	6/6	①②
Zhou (2012)	41/30	SLIT-DM/SCIT-DM	12/12	①
Zou (2014)	28/27/27	PharT/SLIT-DM/SCIT-DM	24/24/24	①②

PharT, pharmacotherapy; SCIT, subcutaneous immunotherapy; SLIT, sublingual immunotherapy; DM, dust mite; GP, grass mix plus pollen extract; ① symptom score; ② adverse reactions. All the studies are RCTs.

### Results of network meta-analysis

#### Network relationship diagram

**Figure 2:**
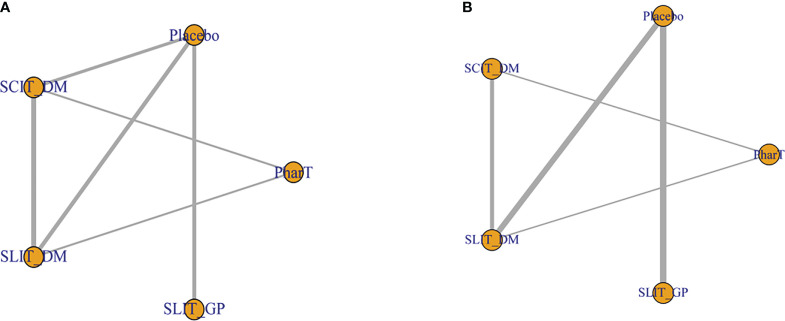
**(A)** Network relationship diagram for symptom score. **(B)** Network relationship diagram for adverse reactions.

The network relationships of the five different interventions in this study (placebo, PharT, SCIT_DM, SLIT_DM, SLIT_GP) are shown in **Figure 2**. Node size represents sample size, and a connector line between nodes indicates a direct comparison between two interventions. Additionally, the thicker the connector line, the more the included studies. The network relationship diagram for symptom scores (**Figure 2A**) suggested a closed loop between placebo, SLIT-DM, and SCIT-DM, as well as between PharT, SLIT-DM, and SCIT-DM, indicating both direct and indirect comparisons between different interventions, whereas the network relationship diagram for adverse reactions (**Figure 2B**) displayed a closed loop between PharT, SLIT-DM, and SCIT-DM, which suggested that there were both direct and indirect comparisons between the three interventions.

#### Consistency check

**Figure 3:**
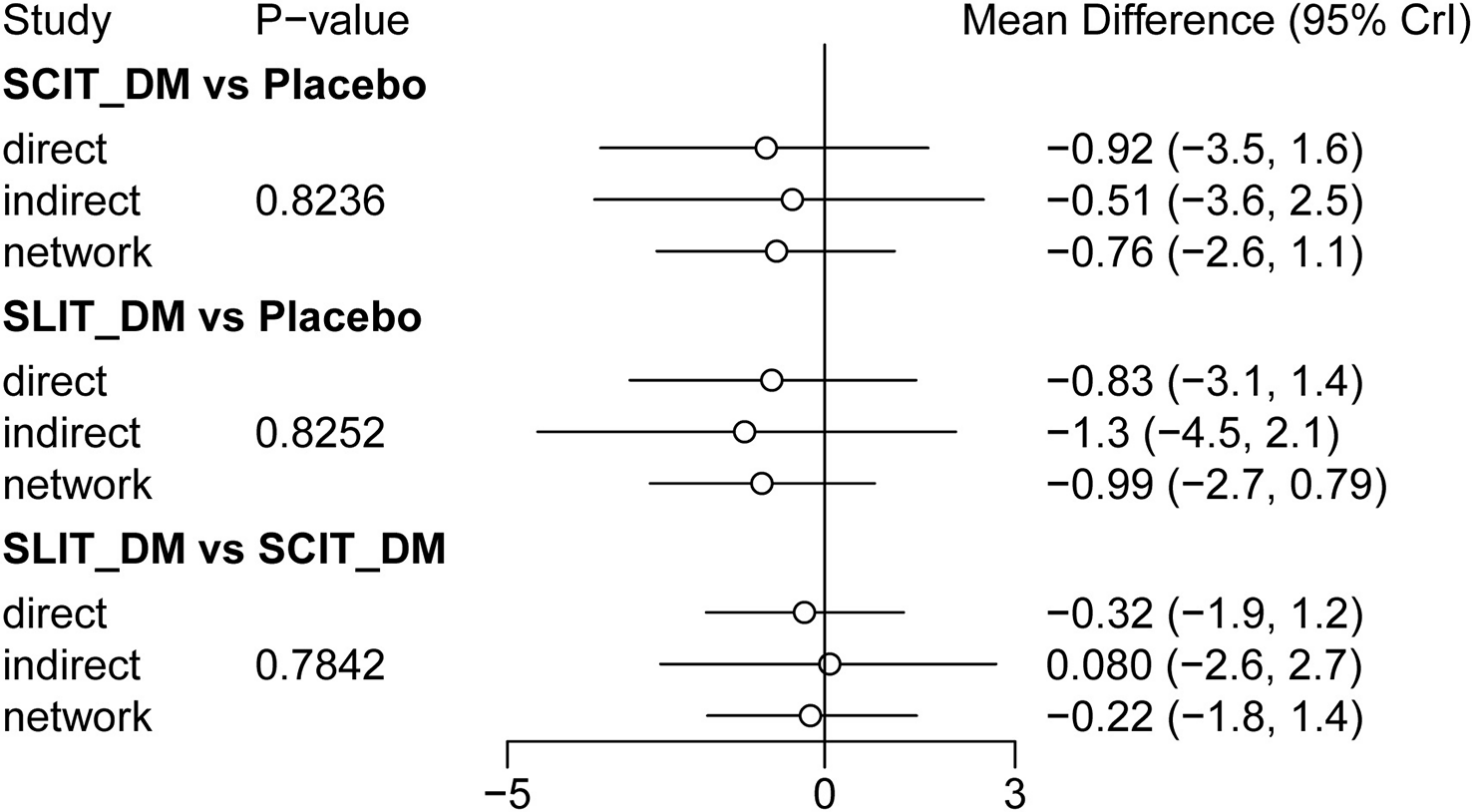
Forest plot.

In this study, node analysis was applied to check the consistency of the comparison results of closed loops in the network relationship diagram. As shown in **Figure 3**, there were no statistical differences between the direct comparison results, indirect comparison results, and combined comparison results of SCIT_DM vs. placebo, SLIT_DM vs. placebo, and SLIT_DM vs. SCIT_DM (*P* > 0.05). Accordingly, the data was of good consistency and could be analyzed using the consistency model.

#### Convergence and stability evaluation

**Figure 4:**
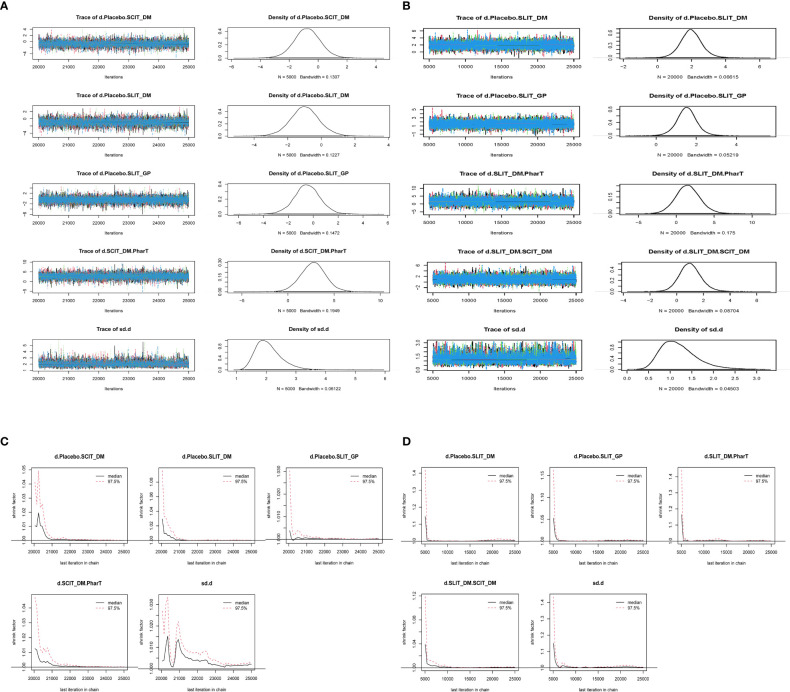
**(A)** Trace and density plots for symptom scores. **(B)** Trace and density plots for adverse reactions. **(C)** Convergent diagnostic plots for symptom scores. **(D)** Convergent diagnostic plots for adverse reactions.

Model convergence and stability were evaluated using convergence diagnostic plots as well as trace and density plots. The trace plots, density plots, and convergence diagnostic plots for symptom scores and adverse reactions are shown in **Figures 4A–D**. The results of trace and density plots for symptom scores and adverse reactions are shown in **Figures 4A, B**. Specifically speaking, the MCMC chain fluctuated steadily with good overlap when the number of iterations exceeded 5,000; when the number of iterations exceeded 20,000, the density plots presented a smooth curve of normal distribution; and the bandwidth value tended to 0 and became stable, demonstrating a strong degree of convergence and good stability. In addition, as shown in **Figures 4C, D**, the potential scale reduced factor (PSRF) values in the convergence diagnostic plots of symptom scores and adverse reactions tended to 1, suggesting that the model convergence was satisfactory.

#### Reticulated meta-analysis and probability ranking

**Figure 5:**
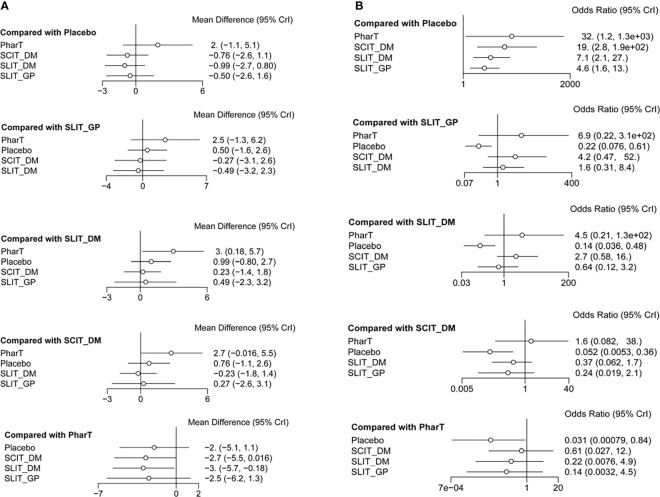
Forest plot for symptom scores **(A)**. Forest plot for adverse reactions **(B)**.

**Figure 6:**
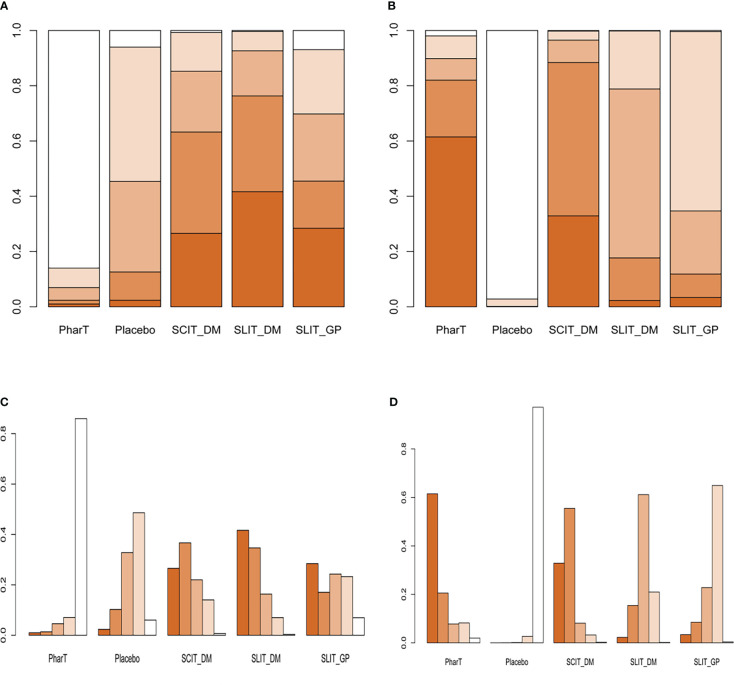
**(A)** Stacking diagram of symptom scores. **(B)** Stacking diagram of adverse reactions. **(C)** Single ranking diagram of symptom scores. **(D)** Single ranking diagram of adverse reactions. PharT, pharmacotherapy; SCIT, subcutaneous immunotherapy; SLIT, sublingual immunotherapy; DM, dust mite; GP, grass mix plus pollen extract.

In this study, a network meta-analysis was performed on the symptom scores and adverse reactions of five different interventions. The forest plots of pairwise comparisons between the five interventions are shown in **Figure 5**, and the league tables are shown in **Table 2** and **Table 3**. Taking symptom score as an indicator, the cumulative probability ranking of each intervention was performed by a Bayesian model. The results showed that the ranking of the five interventions as the best treatment method was SLIT_DM (SUCRA = 0.78), SCIT_DM (SUCRA = 0.69), SLITGP (SUCRA = 0.59), placebo (SUCRA = 0.39), and PharT (SUCRA = 0.06). Therefore, SLIT_DM is the most effective and PharT is the least effective in the treatment of AR, whereas, taking adverse reaction as the indicator, the cumulative probability ranking was PharT, SCIT_DM, SLITGP, SLIT_DM, and placebo. The column stacking diagrams and single ranking diagrams of symptom scores and adverse reactions are shown in **Figures 6A–D**.

**Table 2:** Network meta-analysis of symptom scores.

Interventions	SMD (95% CI)				
	PharT	Placebo	SCIT_DM	SLIT_DM	SLIT_GP
PharT	0	–	–	–	–
Placebo	-1.95 (-5.06, 1.15)	0	–	–	–
SCIT_DM	-2.71 (-5.48, 0.05)	-0.75 (-2.63, 1.10)	0	–	–
SLIT_DM	-2.94 (-5.74, -0.15)	-0.97 (-2.76, 0.82)	-0.21 (-1.88, 1.44)	0	–
SLIT_GP	-2.44 (-6.27, 1.36)	-0.49 (-2.63, 1.64)	0.26 (-2.62, 3.15)	0.47 (-2.29, 3.29)	0

**Table 3:** Network meta-analysis of adverse reactions.

Interventions	OR (95% CI)				
	PharT	Placebo	SCIT_DM	SLIT_DM	SLIT_GP
PharT	0	–	–	–	–
Placebo	3.46 (0.17, 7.14)	0	–	–	–
SCIT_DM	0.49 (-2.50, 3.636)	-2.95 (-5.23, -1.01)	0	–	–
SLIT_DM	1.49 (-1.58, 4.88)	-1.96 (-3.31, -0.73)	0.98 (-0.54, 2.783)	0	–
SLIT_GP	1.93 (-1.50, 5.73)	-1.52 (-2.58, -0.49)	1.43 (-0.75, 3.94)	0.43 (-1.16, 2.13)	0

## Discussion

A total of 22 literature on immunotherapy for AR, including 4,941 patients with five treatment modes, were included in this study. According to the principle of evidence-based medicine, the efficacy of placebo, PharT, SCIT_DM, SLIT_DM, and SLIT_GP was compared by a network meta-analysis in this study. We found that during the follow-up phase, SLIT_DM and SCIT_DM treatment efficacy is superior to SLIT_GP, placebo, and PharT alone. On the cumulative probability of adverse effect metrics, SLIT_ DM was also lower than SCIT_DM and PharT alone and exhibited no significant adverse effects. Therefore, the use of SLIT_DM for AR has the best efficacy, and PharT alone is the worst treatment.

The current clinical treatment of AR is mainly based on pharmacotherapy in China. Glucocorticoids, antihistamines, and leukotriene receptor antagonists are still the main drugs used. Unfortunately, AR is prone to relapsing after stopping such medications, and the actual efficacy of these drugs is difficult to maintain for a long time. Thus, patients with AR experience substantial impairments in their quality of life, as well as an increase in their financial load as a result of long-term medication (14). Unlike conventional pharmacotherapy for symptomatic treatment, SIT is able to stimulate and improve the body’s tolerance to allergic substances in the environment through allergen extracts, so as to achieve the purpose of causal treatment. The most prominent advantage of SIT compared with pharmacotherapy is the maintenance of its long-term efficacy after the end of treatment (31). The traditional SIT for treating AR is SCIT, whereas SLIT emerged afterward. Although the effectiveness of SCIT has been verified for centuries since it was introduced in 1911, its complex operation requires frequent injections for AR patients in medical institutions. In addition, SCIT has the potential for serious adverse reactions or even results in anaphylactic shock. It is these disadvantages that limit the clinical application of SCIT to some extent (32). The network meta-analysis based on the available evidence showed that SLIT_DM was the most effective method, followed by SCIT_DM and SLIT_GP. In China, the two main SLIT allergens used in therapeutic settings are Artemisia annua pollen and Dermatophagoides farinae drops, the latter of which shows long-term effectiveness (33, 34). In terms of clinical efficacy, it is also clear in this study that SLIT_DM is the most effective treatment for AR. Some studies have shown an indirect comparison favoring SLIT over SCIT treatment based on tolerability and safety. SCIT can be associated with allergic reactions (24, 35). In addition, compared with SCIT, SLIT is easy to operate and can be safely self-administered, which results in a high number of local adverse effects of SLIT, such as oral itching, swelling, and throat irritation (7). However, they are usually mild and subside without treatment (35). Meanwhile, systemic side effects of SLIT such as asthma and anaphylaxis are rarer (36). In the present study, we likewise observed that SCIT_DM and PharT alone had more adverse effects than SLIT_DM. A study by Liu et al. (37) similarly confirmed that SLIT had fewer adverse effects than SCIT but that SCIT was to some extent more effective than SLIT. DuBuske et al. (38) found that SLIT tablets were superior to SCIT in terms of safety, but slightly less effective than SCIT. The reason for this occurrence is still due to the inclusion of fewer studies comparing SIT between different modes of administration and the small sample size of some of the included studies, which may have biased the results. However, it is undeniable that all the above studies have shown that SLIT_DM has fewer adverse effects and is safer than SCIT_DM after treating AR.

## Limitations

There are still some limitations in this study (1). There is some heterogeneity between the included studies because randomization, course of treatment, outcome measures, etc., may affect the accuracy of the results. (2) Most studies lack long-term follow-up outcomes, so the long-term efficacy of different interventions on AR cannot be evaluated. (3) Some included studies were blinded to the interventions due to the limited administration routes of the interventions, which may lead to measurement or implementation bias in the analysis results. (4) Of the 22 studies ultimately included, only two had a PharT alone group, and the only outcome indicators were symptom scores and adverse effects, with no additional clinical indicators to assess treatment effects. (5) Based on the shortcomings of the SUCRA rankings themselves, such as failure to consider the magnitude of differences between treatments, clinician familiarity with specific treatments, and the contingent nature of clinical treatments, clinicians using network meta-analysis need to interpret them with caution. Therefore, additional double-blind, multicenter, large-sample, and high-quality RCTs are still required for validation in the future in order to provide a more accurate and objective evidence-based basis for the clinical diagnosis and treatment of AR.

## Conclusion

In summary, this study is the first to compare the clinical efficacy and safety of different immunotherapies for AR through a network meta-analysis. Notably, SLIT_DM has the most prominent overall efficacy. However, the results of this ranking should be viewed with caution in view of the limitations of this study.

## Data availability statement

The original contributions presented in the study are included in the article/supplementary material. Further inquiries can be directed to the corresponding author.

## Author contributions

All the authors participated in the study design. ZJ: conceptualization, formal analysis and writing the original draft. FJ: data curation, methodology, and software. All authors have agreed to the final version.
